# Determinants of Metabolic Syndrome and 5-Year Cardiovascular Risk Estimates among HIV-Positive Individuals from an Indian Tertiary Care Hospital

**DOI:** 10.1155/2020/5019025

**Published:** 2020-10-28

**Authors:** Sneha Deepak Mallya, Sravan Kumar Reddy T, Asha Kamath, Akhilesh Kumar Pandey, Kavitha Saravu

**Affiliations:** ^1^Department of Community Medicine, Kasturba Medical College, Manipal Academy of Higher Education, Manipal, Karnataka 576104, India; ^2^Manipal Centre for Infectious Diseases (MAC ID), Prasanna School of Public Health, Manipal Academy of Higher Education, Manipal, Karnataka 576104, India; ^3^Cardiovascular Health Officer, World Health Organization, Warangal, Telangana 506001, India; ^4^Department of Data Sciences, Prasanna School of Public Health, Manipal Academy of Higher Education, Manipal, Karnataka 576104, India; ^5^Department of Infectious Diseases, Kasturba Medical College, Manipal Academy of Higher Education, Manipal, Karnataka 576104, India

## Abstract

Longer survival due to use of antiretroviral therapy (ART) has made human immunodeficiency virus- (HIV-) infected individuals prone to chronic diseases such as diabetes, hypertension, and cardiovascular diseases (CVD). Metabolic syndrome (MS), a constellation of risk factors which increase chances of the cardiovascular disease and diabetes, can increase the morbidity and mortality among this population. Hence, the present study was conducted with the objectives of estimating the prevalence and determinants of MS among ART naïve and ART-treated patients and assess their 5-year CVD risk using the reduced version of Data Collection on Adverse Effects of Anti-HIV Drugs (D : A : D) risk prediction model (D : A : D(R)). This hospital-based cross-sectional study included 182 adults aged ≥ 18 years. MS was defined using the National Cholesterol Education Program-Adult Treatment Panel-3 (NCEP ATP-3) criteria. Univariate and multivariate logistic regressions were done to identify the factors associated with MS. Prevalence of MS was 40.1% (95% confidence interval (CI) = 33.0%–47.2%). About 24.7% of the participants had at least a single criterion for MS. Age >45 years (adjusted odds ratio (AOR) = 2.3; 95% CI = 1.1–4.9, *p* < 0.018) and body mass index (BMI) > 23 kg/m^2^ (AOR = 6.4; 95% CI = 3.1–13.1, *p* < 0.001) were positively associated with MS, whereas daily consumption of high sugar items was inversely associated (AOR = 0.2; 95% CI = 0.1–0.5, *p* < 0.001). More than 50% of the participants were found to have moderate or high 5-year CVD risk. Observed prevalence of MS among HIV patients was higher than other studies done in India. Considering a sizeable number of participants to be having moderate to high CVD risk, culturally appropriate lifestyle interventions need to be planned.

## 1. Introduction

Human immunodeficiency virus (HIV) continues to be a major public health issue with 36.7 million people living with HIV globally and 53% of them receiving ART [1]. With the widespread use of ART, the survival rates have greatly improved among HIV-infected individuals [2]. However, there have been reports of long-term ART-related increase in cardiovascular risk among HIV-positive patients [3]. Also, CVD has become an important cause of death among HIV patients [4]. A number of metabolic abnormalities have been detected in HIV patients including dyslipidemia and insulin resistance which can increase the CVD risk [5]. A complex interaction between the host's advancing age, virus, inflammatory process, and ART use has been described as the underlying mechanism for the increased CVD risk among HIV-infected patients [6].

A quarter of deaths due to noncommunicable diseases were attributed to CVDs in India in the year 2014 [7]. Metabolic syndrome is a constellation of risk factors which can increase an individual's risk of developing atherosclerotic cardiovascular disease (ASCVD) [8]. Hence, metabolic syndrome as an entity can greatly contribute to enhanced CVD burden. While there exist some studies addressing metabolic syndrome in HIV-infected population among Indians, the estimates vary [9–11]; a few studies are devoid of data on key cardiovascular risk factors such as tobacco, alcohol, and physical activity. With clear lacunae of such studies from India, this study was planned to estimate the prevalence and identify the factors associated with metabolic syndrome. With widespread availability of ART under the National AIDS (Acquired Immunodeficiency Syndrome) Control Program (NACP) and also the private sector institutions in India, the impact of CVD complications among HIV-infected individuals may undermine the success of health benefits achieved through ART use. Early diagnosis and prevention of these conditions may help to reduce the morbidity and mortality associated with CVD complications.

## 2. Materials and Methods

A cross-sectional study was conducted among HIV-positive ART-naïve individuals and those receiving ART at a tertiary care teaching hospital in southern India during March–June 2017. The study included all the HIV-positive ART-treated and ART-naïve adult males and nonpregnant females aged ≥18 years, who visited the hospital during the study period.

A clearance was obtained from the Institutional Ethics Committee of Kasturba Medical College and Kasturba Hospital, Manipal, before the commencement of the study (IEC 26/2017). A written informed consent was obtained from the participants. Details including sociodemographic characteristics and behavioral factors such as diet, tobacco, and alcohol consumption were collected using a structured questionnaire (Supplementary Appendix 1). An enquiry was made regarding frequency of consumption of high salt items (extra table salt, pickle, salted fish, salted nuts, salt in salad, and salted snacks), high sugar items (sweets, soft drinks, jam, cakes, honey, ice cream, and extra table sugar), high fat items (cakes, sweets, ice cream, ghee, dalda, butter, cream, chocolates, fried food items, cookies, red meat, and junk food items), fish, and fruits and vegetables. The frequency of consumption of various food items was classified as daily, 2-3 times per week, at least once a week, and rarely. Current user of tobacco or alcohol was defined as daily or occasional consumption during the current year, and past user was defined as one who has quit for more than a year. A person who used tobacco or alcohol either currently or in the past was considered to have “ever used” any substance. Physical activity was estimated using the International Physical Activity Questionnaire (IPAQ), and the participants were classified as inactive, minimally active, and health-enhancing physical activity (HEPA) [12]. The 5-year cardiovascular risk was estimated among individuals by a Data Collection on Adverse Effects of Anti-HIV Drugs [D : A : D (R)] risk prediction model using a web-based calculator (https://www.chip.dk/Tools-Standards/Clinical-risk-scores). This tool is based on a reduced model and estimates the risk of an individual developing cardiovascular disease (CVD) within the next 5 years. The D : A : D (*R*) does not include ART as a parameter, and it requires the following information: gender, age, smoking status, diabetes (diagnosis or on antidiabetic treatment), family CVD history, systolic blood pressure (SBP), total cholesterol, high-density lipoprotein (HDL), and cluster of differentiation 4 (CD4) count. The composite CVD outcome includes myocardial infarction, stroke, invasive coronary artery procedure (including coronary artery by-pass or angioplasty and carotid artery endarterectomy), or death from coronary heart disease. This D : A : D (*R*) model is valid for HIV-infected individuals aged 18–75 years. The 5-year risk of CVD was classified as low (< 1%), moderate (1 to 5%), high (5 to 10%), or very high (>10%) [13].

Anthropometric measures such as weight (kg), height (cm), and waist circumference (cm) were measured. Body mass index (BMI) was computed and classified based on Indian classification [14]. Two blood pressure (BP) readings were taken through a digital sphygmomanometer, in sitting position in the right arm, 10 minutes apart, and an average of two readings was considered. Hypertension was defined as SBP/DBP of ≥140/90 for those aged <60 years and ≥150/90 for individuals aged above 60 years [15]. Diabetes was defined as fasting plasma glucose (FPG) ≥126 mg/dl and or postprandial plasma glucose ≥200 mg/dl [16]. Hypothyroidism was defined as serum triiodothyronine level <0.80 ng/ml or serum thyroxine level <5.1 micrograms/dl and or serum thyroid-stimulating hormone level >4.2 microinternational units/ml [17]. National Cholesterol Education Program-Adult Treatment Panel (NCEP-ATP3) 2005 revision criteria were used to define MS which were as follows: abdominal obesity (waist circumference ≥ 90 cm for Asian men or ≥80 cm for Asian women), fasting triglycerides (TG) ≥ 150 mg/dl or drug treatment for elevated triglycerides, HDL cholesterol (HDL-C) ≤ 40 mg/dl for men or ≤50 mg/dl for women or on drug treatment for reduced HDL-C, systolic/diastolic blood pressure(SBP/DBP) ≥ 130/85 mmHg or receiving drug treatment, and FPG ≥ 100 mg/dl or drug treatment for elevated glucose [18]. Asian cutoffs for abdominal obesity were taken [19, 20]. Participants having three or more of the above criteria were considered to have MS.

### 2.1. Statistical Analysis

The data were entered and analyzed using the Statistical Package for Social Sciences (SPSS) version 15. Continuous variables were summarized using mean (standard deviation (SD)) or median (interquartile range (IQR)), and categorical variables have been expressed as percentages. The chi-square test and independent-sample *t*-test were used to find out association between categorical and continuous variables, respectively. The Mann–Whitney test was used to compare mean DAD scores. Univariate and multivariate logistic regressions were done to identify the factors associated with MS and unadjusted and adjusted odds ratio (OR), respectively, with 95% confidence intervals (CI) have been reported. The variables which were significant in univariate analysis were included in the multivariate analysis. A *p* value of <0.05 was considered to be statistically significant.

## 3. Results

**Figure 1:**
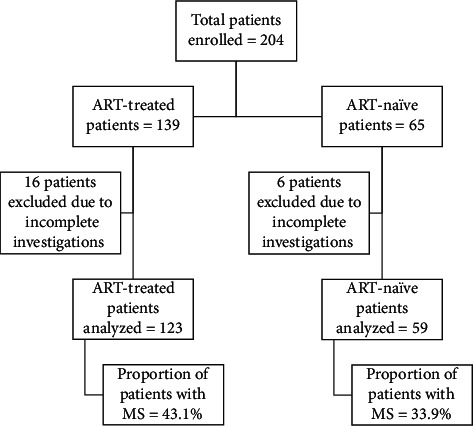
Flowchart showing recruitment of study participants.

The study included a total of 182 (123 ART-treated and 59 ART-naïve) participants who visited the outpatient facility of the department of medicine or hospitalized as an inpatient during the months of March to June 2017. The recruitment of study participants is shown in Figure 1.

As shown in Table 1, non-nucleoside reverse transcriptase inhibitor- (NNRTI-) based ART regimen was administered to 75.4% of the participants, while 24.6% were on protease inhibitor- (PI-) based regimen.

A quarter of the participants (24.7%) had at least one criterion for MS, and 3.8% of the participants had all the five criteria for MS. The overall prevalence of MS among participants was observed to be 40.1% (95% CI = 33.0%–47.2%) with a higher prevalence among individuals on ART as compared to ART-naïve individuals (43.1% vs 33.9%). However, this difference was not statistically significant (*p*=0.23). There was no difference in the prevalence of MS across the two genders (males = 40.4% vs females = 40.3%, *p*=0.96).

The most common abnormal MS component observed was low HDL (68.1%), whilst the least common was BP ≥130/85 or being on antihypertensives (28.6%).

Distribution of the overall morbidities among the participants was diabetes (15.9%), hypertension (20.3%), and hypothyroidism (5.5%). As shown in Table 2, the lipid abnormalities were higher in ART-naïve individuals and the other three components of MS were present to a greater extent among individuals on ART. The above differences with respect to various criteria of metabolic syndrome were found to be statistically significant among ART-naïve and ART-treated patients except for elevated TG levels. Low HDL values in ART naive could be because of them being more sedentary compared to people on ART.

Females had higher prevalence of abdominal obesity (49.3% vs 27.8%, *p*=0.004) and low HDL (79.1% vs 61.7%, *p*=0.015) as compared to males. Males displayed a higher frequency of elevated TG (48.7% vs 46.3%, *p*=0.75), BP (32.2% vs 22.4%, *p*=0.15), and fasting blood sugar (53.9% vs 35.8%, *p*=0.018) against females.

Among the study participants, 63.2% were male. Univariate analysis to find out the association between MS and sociodemographic, disease-related, and lifestyle factors has been shown in Table 3. It was observed that age > 45 years., BMI > 23 kg/m^2^, duration since HIV infection > 36 months, being on ART > 36 months, and family history of diabetes, hypertension, and CVD were positively associated with MS. Consumption of high sugar and salt items on a daily basis or 2-3 times/week or once a week were inversely associated with MS.

 The variables which were significant in univariate analysis were included in multivariate analysis. As shown in Table 4, multivariate analysis found that age > 45 years and BMI > 23 kg/m^2^ were independently associated with MS, whereas daily high-sugar item consumption showed an inverse association.

**Figure 2:**
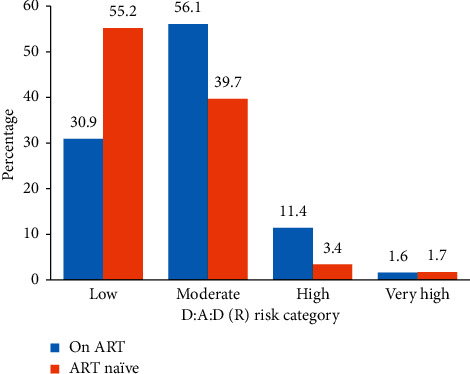
D : A : D(R) 5-year risk of CVD development by ART status (*n* = 181*∗*). *∗*D : A : D risk score could not be calculated for a participant as the age was above 80 years.

Figure 2 shows the 5-year CVD risk among the study participants stratified by ART status. The overall median (IQR) D : A : D risk score for the study population was 1.4 (0.6,2.8). Following was the distribution of 5-year CVD risk among all participants: low: 70 (38.7%), moderate: 92 (50.8%), high: 16 (8.8%), and very high: 3 (1.7%). A higher proportion of the participants belonged to moderate and high CVD risk groups in ART-treated category as compared to ART naïve, and this difference was found to be statistically significant (*p* < 0.001). The median D : A : D score was significantly higher among males as compared to females (1.7 vs 0.8, *p* < 0.001). A significant difference was also noted between median D : A : D scores of the two groups, i.e. ART-treated vs ART-naïve individuals (1.7 vs 1.0, *p* < 0.009).

## 4. Discussion

The prevalence of MS in the present study was 40.1%. This is higher when compared to studies done elsewhere in India in which prevalence ranged from 19.8 to 26.6% [9–11]. Studies conducted outside India have reported a prevalence of MS from the lowest of 15.6% to the highest of 48.3% [21–29]. The prevalence of MS in the current HIV-infected participants is higher as compared to uninfected individuals in India in which the prevalence ranged from 18.4 to 30.9% [30, 31]. While most of the studies have used NCEP-ATP 3 criteria, few studies have used International Diabetes Federation (IDF) criteria for assessment of MS. Apart from the criteria used for assessment, this disparity may be attributed to the variation in prevalence of MS components among the patient population studied. Additionally, factors such as the type of study population selected (ART-naïve or ART-treated) and the type and duration of exposure to ART may be contributing factors for this observed difference.

The current study did not observe any statistically significant difference in MS prevalence between ART-treated and ART-naïve individuals. However, many studies have reported prevalence of MS to be higher among patients on ART as compared to their ART-naïve counterparts [9, 11, 29]. Results from a Nigerian study also reported that there was no increase in MS among the ART-treated group [27]. This may be due to the reason that only 0.6% of the study population received PI-based ART regimen, which are known to be associated with metabolic changes. The current study did not observe such association as PI-based regimen (ritonavir and atazanavir) was used among only 31 participants, and hence, did not have adequate power to detect this association.

The current study found a quarter of the participants to be having at least one risk factor for MS. The proportion of participants with at least one abnormal MS component ranged from one-third to almost two-thirds of the study population among various other studies [10, 32]. There was a wide variation in the type of predominant abnormal component among different studies. Similar to the findings of the present study, other studies from India and abroad [10, 11, 22, 25] have reported low HDL as the most common abnormal component of MS among HIV-positive individuals. While the present study found that BP > 130/85 mm Hg or being on antihypertensive medication to be the least common abnormal component of MS, the study from Southeast Nigeria [27] observed it to be the most dominant component. Females in the study population had higher prevalence of low HDL cholesterol and abdominal obesity, and this may be a reflection of real differences pertinent to this population of coastal Karnataka and deserves further investigation.

The overall median 5-year CVD risk observed in the present study was higher than the study findings from Cameroon (0.6% (0.3–1.3)) in which 80% of participants were females [33]. As 63% of study population were males who are traditionally known to have higher CVD risk, the median score is higher in the present study. The study also found that median (IQR) 5-year CVD risk was higher among males in comparison to females (1.4% (0.8–2.7) vs 0.5% (0.3–0.9), *p* < 0.001), which is akin to findings of the present study [33]. While in the current study 38.7% of participants had low 5-year CVD risk, a study from Pune found that 63.5% of the study participants (median age = 40 years) had low risk [34]. Another study from Brazil found that two-thirds of the HIV-infected participants (mean age = 36.8 years) to be having low risk (74.2%) [22]. Participants in the both the aforementioned studies were younger as compared to our study population which may be the reason for predominance of the low-risk category.

The present study found that age > 45 years and high BMI to be positively associated with MS. Association between these traditional CVD risk factors and MS have been demonstrated in several studies among HIV-positive individuals [23, 24, 28, 35]. While current study did not find any gender disparity in MS prevalence, Alvarez [24] and Berhane et al. [26] observed a female preponderance.

Studies by Pongthananikorn et al. from India [36] and Tiozzo et al. from United States of America [37] found a positive association between high sugar item consumption and MS; however, the present study found an inverse association between the two. This may be attributed to the fact that individuals with MS had higher frequency of diabetes and hypertension and, hence, may have reduced the consumption of sugar and salt, respectively.

Our study systematically dissected all components of metabolic syndrome in HIV-infected individuals in India. We further estimated their 5-year cardiovascular risk. Our study has highlighted the importance of higher BMI, a modifiable risk factor for MS, which could guide future preventive interventions. However, our study has certain limitations. As this study was carried out in a tertiary referral center, MS prevalence in the study setting may not be generalizable to the general HIV+ population. The information collected on tobacco, alcohol consumption, and dietary factors was minimal, and this might have affected the results. Larger prospective studies are necessary to provide better understanding of the factors associated with MS.

## 5. Conclusion

Prevalence of MS is high among the HIV-infected study participants. Predictive factors for MS in this study were age and BMI, both being traditional risk factors. None of the HIV-related factors such as duration of infection, exposure to ART, and CD4 count were associated with presence of MS in this population. More than half of the study participants had moderate or high risk of development of CVD in the next five years. It is of utmost importance to develop specific interventions to prevent, diagnose, and treat MS among HIV-infected patients to reduce their long-term morbidity and mortality. Early identification and instituting appropriate management will help to reduce future complications and mortality due to CVD.

## Figures and Tables

**Table 1:** Details of the study participants according to class of ART received (*n* = 123).

Drug class	Name of the drug	*n* (%)
NNTRI	Efavirenz	82 (66.7)
	Nevirapine	13 (10.6)

NRTI	Tenofovir	97 (78.9)
	Emtricitabine	65 (52.8)
	Lamivudine	54 (43.9)
	Zidovudine	26 (21.1)
	Abacavir	4 (3.3)

PI	Atazanavir/ritonavir	28 (15.4)
	Darunavir/ritonavir	2 (1.6)
	Lopinavir/ritonavir	1 (0.8)

NNRTI, non-nucleoside reverse transcriptase inhibitors; NRTIs, nucleoside reverse transcriptase inhibitors; PI, protease inhibitor.

**Table 2:** Various criteria for metabolic syndrome by ART status (*n* = 182).

Criteria for MS	On ART (*n* = 123)	ART naïve (*n* = 59)	*p* value
	*n* (%)	*n* (%)	
Presence of abdominal obesity	52 (42.3)	13 (22.0)	0.008
Elevated fasting TG or drug treatment for elevated TG	56 (45.5)	31 (52.5)	0.37
Low HDL-C or on drug treatment for reduced HDL-C	73 (59.3)	51 (86.4)	<0.001
Elevated SBP/DBP ≥ 130/85 mmHg or receiving drug treatment	42 (34.1)	10 (16.9)	0.01
Elevated FPG or drug treatment for elevated glucose	66 (53.7)	20 (33.9)	0.01

TG, triglycerides; HDL-C, high-density lipoprotein cholesterol; SBP, systolic blood pressure; DBP, diastolic blood pressure; FPG, fasting plasma glucose.

**Table 3:** Factors associated with MS on univariate analysis (*n* = 182).

Variable	Category	MS present (*n* = 73)	MS absent (*n* = 109)	OR with 95% CI	*p* value
*Background characteristics*					
Age in years	≤45	23 (31.5)	56 (51.4)	1	—
	>45	50 (68.5)	53 (48.6)	2.2 (1.2–4.2)	0.009

Gender	Male	46 (63.0)	69 (63.3)	0.9 (0.5–1.8)	0.96
	Female	27 (37.0)	40 (36.7)	1	—

Marital status	Ever married	71 (97.3)	98 (89.9)	1	—
	Single	2 (2.7)	11 (10.1)	0.2 (0.0–1.1)	0.07

Education	Up to 7 years of schooling	19 (26.0)	30 (27.5)	0.9 (0.4–1.8)	0.82
	>7 years of schooling	54 (74.0)	79 (72.5)	1	—

Occupation	Employed	44 (60.3)	78 (71.6)	1	—
	Homemaker/unemployed	29 (39.7)	31 (28.4)	1.6 (0.8–3.1)	0.11

Average monthly income	(Median (IQR))	4000 (2500, 7083)	4000 (2500, 6667)	1.0 (1.0–1.0)	0.55
*Disease- and treatment-related factors*					
Duration of HIV infection	≤36 months	32 (43.8)	66 (60.6)	1	—
	>36 months	41 (56.2)	43 (39.4)	1.0 (1.0–3.5)	0.02

Recent CD4 count in cells/mm^3^	≤500	56 (76.7)	89 (81.7)	0.7 (0.3–1.5)	0.41
	>500	17 (23.3)	20 (18.3)	1	—

Treatment status	On ART	53 (72.6)	70 (64.2)	1.4 (0.7–2.8)	0.23
	ART naïve	20 (27.4)	39 (35.8)	1	—

Duration of ART (*n* = 123)	≤36 months	13 (24.5)	31 (44.3)	1	—
	>36 months	40 (75.5)	39 (55.7)	2.4 (1.1–5.3)	0.02

ART regimen	NNRTI based	41 (56.2)	50 (45.9)	1.5 (0.8–3.1)	0.17
	PI based	12 (16.4)	20 (18.3)	1.1 (0.4–2.8)	0.73
	ART naive	20 (27.4)	39 (35.8)	1	—

*Lifestyle factors*					
BMI	Normal (≤23 kg/m^2^)	24 (32.9)	81 (74.3)	1	—
	Overweight and obese (>23 kg/m^2^)	49 (67.1)	28 (25.7)	5.9 (3.0–11.3)	< 0.001

Physical activity	Inactive	35 (32.1)	22 (30.1)	0.9 (0.2–3.7)	0.93
	Minimally active	68 (62.4)	47 (64.4)	1.0 (0.2–3.8)	0.95
	HEPA	6 (5.5)	4 (5.5)	1	—

Tobacco consumption	Ever	11 (15.1)	21 (19.3)	0.7 (0.3–1.6)	0.46
	Never	62 (84.9)	88 (80.7)	1	—

Alcohol consumption	Ever	19 (26.0)	30 (27.5)	0.9 (0.4–1.8)	0.82
	Never	54 (74.0)	79 (72.5)	1	—

High-fat item consumption	Daily	8 (11.0)	23 (21.1)	0.4 (0.1–1.1)	0.09
	2-3 times/week to once/week.	39 (53.4)	53 (48.6)	0.9 (0.4–1.8)	0.83
	No/rarely	26 (35.6)	33 (30.3)	1	—

High-sugar item consumption	Daily	24 (32.9)	63 (57.8)	0.2 (0.09–0.4)	< 0.001
	2-3 times/week to once/week	19 (26.0)	30 (27.5)	0.3 (0.1–0.7)	0.011
	No/rarely	30 (41.1)	16 (14.7)	1	—

High-salt item consumption	Daily	29 (39.7)	56 (51.4)	0.3 (0.1–0.7)	0.01
	2-3 times/week to once/week	21 (28.8)	37 (33.7)	0.3 (0.1–0.9)	0.02
	No/rarely	23 (31.5)	16 (14.7)	1	—

Fish consumption	Daily	9 (12.3)	7 (6.4)	1	—
	2-3 times/week to once/week	33 (45.2)	36 (33.0)	0.7 (0.2–2.1)	0.54
	No/rarely	31 (42.5)	66 (60.6)	0.3 (0.1–1.0)	0.06

Vegetable consumption	Daily	71 (97.3)	108 (99.1)	1	—
	2-3 times/week	2 (2.7)	1 (0.9)	3.0 (0.2–34.1)	0.36

Family history of hypertension	Present	25 (34.2)	20 (18.3)	2.3 (1.6–4.5)	0.01
	Absent	48 (65.8)	89 (81.7)	1	—

Family history of DM	Present	26 (35.6)	27 (24.8)	1.6 (0.8–3.2)	0.11
	Absent	47 (64.4)	82 (75.2)	1	—

Family history of CVD	Present	7 (9.6)	2 (1.8)	5.6 (1.1–28.1)	0.03
	Absent	66 (90.4)	107 (98.2)	1	—

HIV, human immunodeficiency virus; CD4, cluster of differentiation 4; ART, anti-retroviral therapy; NNRTI, non-nucleoside reverse transcriptase inhibitor; PI, protease inhibitor; BMI, body mass index; HEPA, health enhancing physical activity; CVD, cardiovascular disease;

**Table 4:** Factors associated with MS on multivariate analysis (*n* = 182).

Variable		OR with 95% CI	*p* value
Age	≤45 years	1	0.01
	>45 years	2.3 (1.1–4.9)	

BMI	Normal (≤23 kg/m^2^)	1	<0.001
	Overweight and obese (>23 kg/m^2^)	6.4 (3.1–13.1)	

High-sugar item consumption	No/rarely	1	0.001
	Daily	0.2 (0.1–0.5)	

BMI, body mass index.

## Data Availability

The data used to support the findings of this study are available from the corresponding upon request.

## Supplementary Materials

Supplementary Materials Supplementary Appendix 1: it consists of study tool with details pertaining to sociodemographic characteristics, disease-related information, behavioral factors such as diet, tobacco, and alcohol consumption, physical examination, and biochemical investigations.
