# Supplementary material for: Determinants of Metabolic Syndrome and 5-Year Cardiovascular Risk Estimates among HIV-Positive Individuals from an Indian Tertiary Care Hospital
Source: AIDS Res Treat. 2020 Oct 28;2020:5019025. doi: 10.1155/2020/5019025 (PMC7641704; doi:10.1155/2020/5019025)
Supplement: Supplementary Materials — Supplementary Appendix 1: it consists of study tool with details pertaining to sociodemographic characteristics, disease-related information, behavioral factors such as diet, tobacco, and alcohol consumption, physical examination, and biochemical investigations. [file 5019025.f1.docx]

**Questionnaire**

Proforma no.

1. Age:
2. Gender :
3. Date of collection of data:
4. Educational status:
5. Marital status: Married/Single/Separated/Divorced
6. Occupation:
7. Monthly income:
8. Category of the study participant: On ART/ ART naïve
9. Do you have any of the following diagnosed comorbidity?

Hypertension/Diabetes/Cardiovascular disease/Hypothyroidism/Any other

1. Do you have family history of any of the following?

- Cardiovascular events :Yes /No
- Hypertension: Yes /No
- Diabetes: Yes /No

1. Disease related variables

| Type of HIV infection (HIV1/HIV2/Both) |  |
| --- | --- |
| Duration since diagnosis in months |  |
| Most recent CD4 at with date |  |

1. Treatment related variables:

| Current ART regimen: | Duration since current ART regimen: |
| --- | --- |
| ART regimen used in the past: | Duration of regimen used in the past: |

1. Diet history

| Consumption of: | Yes /No | Frequency of intake | | | |
| --- | --- | --- | --- | --- | --- |
|  |  | Daily | 2-3 times/week | weekly | Rarely |
| High Fat items |  |  |  |  |  |
| High Salt items |  |  |  |  |  |
| High Sugar items |  |  |  |  |  |
| Fish |  |  |  |  |  |
| Fruits & vegetables |  |  |  |  |  |

| (HIGH FAT ITEMS eg. Cakes, Puddings,Pastries, Sweets, Icecream, Ghee, Dalda, Butter, Cream, Chocolates, Fried Food Items, Chips, Cookies, Red Meat, Junk Food Items) Any Other:. ………………..  (HIGH SALT ITEMS eg. Extra Table Salt, Pickles,Salted Fish, Salted Nuts, Salt In Salad, Salted Snacks,Namkeen, Chips) Any Other:………………………………..  (HIGH SUGAR ITEMS eg. Sweets , Soft Drinks, Jam , Cakes, Pastries, Pudding, Honey, Icecream, Extra Table Sugar) Any Other: …………………………….. |
| --- |

1. Habits:

| Habit | 1.Yes 2.No | Current/ Past | Quantity per day | Age of initiation | Age when stopped |
| --- | --- | --- | --- | --- | --- |
| Smokeless tobacco use |  |  |  |  |  |
| Alcohol |  |  |  |  |  |
| Smoking |  |  |  |  |  |

1. Details of physical examination and Investigations

| FBS | PPBS | TG |  |
| --- | --- | --- | --- |
| Total cholesterol | HDL | LDL |  |
| Weight in kg |  | Waist circumference in cm |  |
| Height in cm |  | Hip circumference in cm |  |
| Body Mass Index |  | Waist:Hip ratio |  |
| Blood pressure reading 1 |  | Blood pressure reading 2 |  |
